# Supplementary material for: NeuroQuery, comprehensive meta-analysis of human brain mapping
Source: eLife. 2020 Mar 4;9:e53385. doi: 10.7554/eLife.53385 (PMC7164961; doi:10.7554/eLife.53385)
Supplement: Source data 1. [file elife-53385-data1.zip › neuroquery_elife_53385_additional_data/figure_08/ibc_documentation.pdf]

Individual Brain Charting project

Documentation

Individual Brain Charting

Individual Brain Charting

Ana Luísa Pinho
Juan Jesús Torre
Bertrand Thirion

March 2019

This document presents the organization of the *Individual Brain Charting* (IBC) dataset.

Contents

1 Data organization 4

2 Acquisition parameters 5

2.1 2D Spin-Echo . . . 5

2.2 Diffusion . . . 6

2.3 T1 . . . 7

2.3.1 T1 relaxometry . . . 9

2.4 T2 . . . 10

2.4.1 T2 relaxometry . . . 11

2.5 BOLD-contrast EPI sequences . . . 13

3 Tasks 14

3.1 ARCHI tasks . . . 14

3.1.1 ARCHI Standard . . . 14

3.1.2 ARCHI Spatial . . . 16

3.1.3 ARCHI Social . . . 17

3.1.4 ARCHI Emotional . . . 18

3.2 HCP tasks . . . 19

3.2.1 HCP Emotion . . . 19

3.2.2 HCP Gambling . . . 20

3.2.3 HCP Motor . . . 20

3.2.4 HCP Language . . . 21

3.2.5 HCP Relational . . . 21

3.2.6 HCP Social . . . 22

3.2.7 HCP Working Memory . . . 22

3.3 RSVP Language task . . . 24

4 Processing pipeline 26

4.1 Preprocessing pipeline . . . 26

4.1.1 FMRI Model Specification . . . 27

4.1.2 Model Estimation . . . 27

References 28

A Experimental-design diagrams 30

B Acquisition table 34

1 Data organization

The tree structure of the IBC dataset follows BIDS Specification (http://bids.neuroimaging.io/), as in Figure 1.

- The identifiers of the 13 participants are "sub-01", "sub-02", "sub-04", ..., "sub-15".
- The acquisitions are organized in sessions ("ses-00", "ses-01", ..., "ses-20", etc.).
- Within each session, data is divided according to modality: 'anat', 'dwi', 'fmap', 'func'.
- For each modality, files are stored in .nii.gz format, with a name that recapitulates subject, session and modality together with meta-information stored in .tsv and .json files.

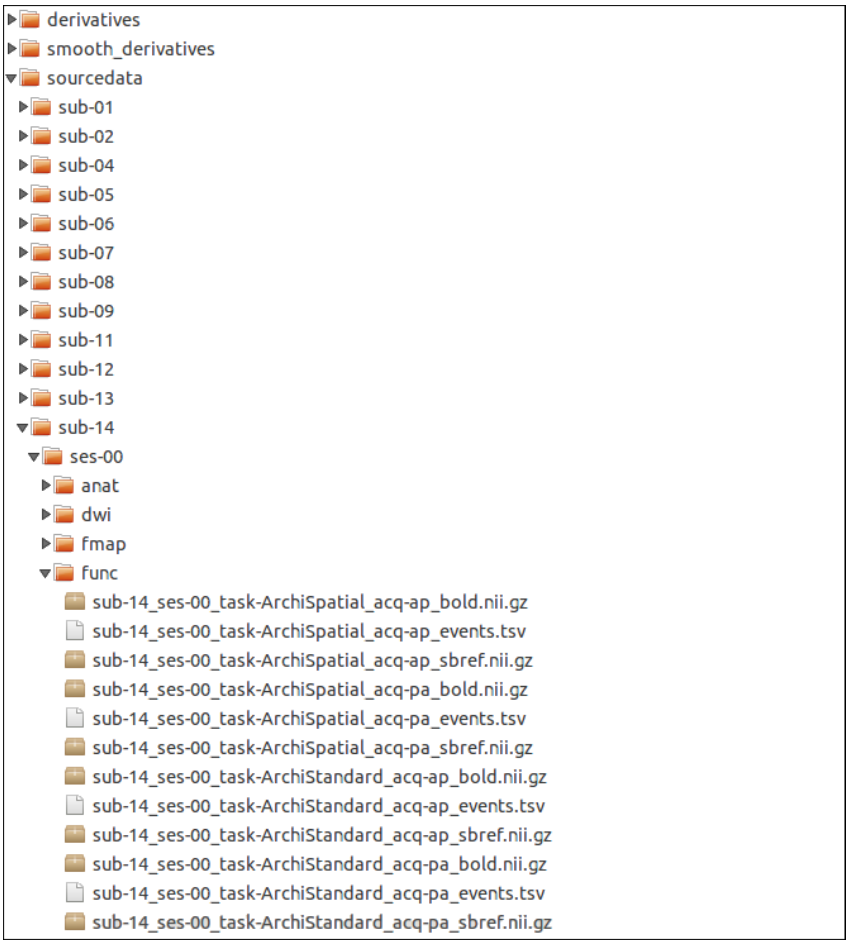

Figure 1: Imaging modalities employed in each session.

2 Acquisition parameters

This section contains tables detailing the acquisition parameters of all the different neuroimaging modalities used in the IBC project. Figure 2 depicts the temporal sequence of runs in terms of neuroimaging modalities within sessions:

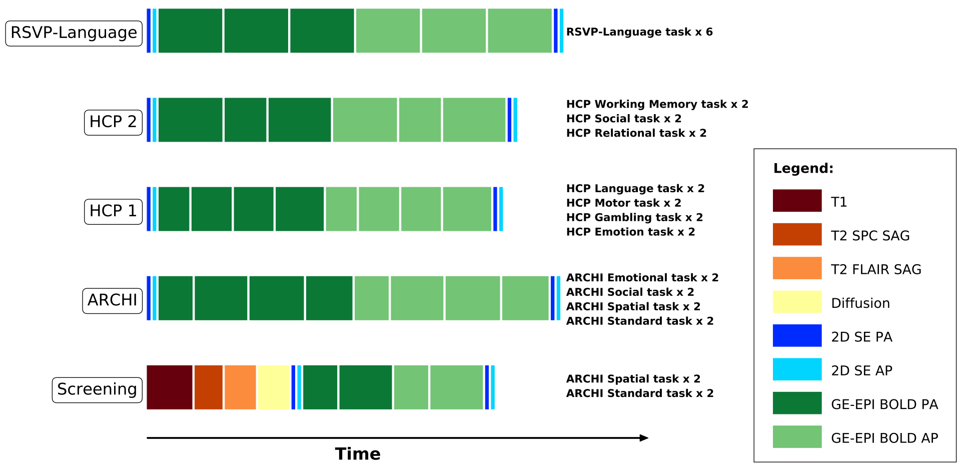

Figure 2: Temporal sequence of runs in terms of neuroimaging modalities for all sessions of the first data release.

2.1 2D Spin-Echo

The 2D Spin-Echo maps are used to obtain a model of distortions for EPI images: a pair of AP/PA images are acquired jointly with each EPI (BOLD or diffusion-weighted) acquisition.

Parameter	Value
Sequence	Spin-echo EPI
TR	9500 ms
TE	37.00 ms
Flip angle	90 deg
Refocusing flip angle	180 deg
FOV	240 x 240 mm
Matrix	128 x 128
Slice thickness	1.30 mm
Multiband accel. factor	1
Echo spacing	0,71 ms
BW	1598 Hz/Px
Phase partial Fourier	6/8
b-values	0 s/mm^2

Table 1: Acquisition parameters for Spin-Echo

2.2 Diffusion

Three types of diffusion sequences were employed in three different runs, respectively:

- High-resolution (1.3mm isotropic, 60 directions) acquisitions with $B = 1500$ or $B = 3000$.

Parameter	Value
Sequence	diff_dw60_TE76
TR	7000 ms
TE	76 ms
Flip angle	90 deg
Refocusing flip angle	180 deg
FOV	240 x 240 mm
Matrix	128 x 128
Slice thickness	1.30 mm, 112 slices, 1.30 mm isotropic
Multiband accel. factor	2
Echo spacing	0.71 ms
BW	1598 Hz/Px
Phase partial Fourier	6/8
b-values	[1500, 3000] s/mm^2

Table 2: Acquisition parameters for high-resolution diffusion imaging

- Multi-shell (1.3mm isotropic, 20 directions) acquisitions for multiple B-values ranging from 300 to 3000 in steps of 300.

Parameter	Value
Sequence	diff_dw26_TE76
TR	7000 ms
TE	76 ms
Flip angle	90 deg
Refocusing flip angle	180 deg
FOV	240 x 240 mm
Matrix	128 x 128
Slice thickness	1.30 mm, 112 slices, 1.30 mm isotropic
Multiband accel. factor	2
Echo spacing	0.71 ms
BW	1598 Hz/Px
Phase partial Fourier	6/8
b-values	[0, 300, 600, 900, 1200, 1500, 1800, 2100, 1400, 2700, 3000] s/mm^2

Table 3: Acquisition parameters for multi-shell diffusion imaging

- Two low-resolution acquisitions (2mm, 20 directions) used for screening.

Parameter	Value	Parameter	Value
Sequence	diff_screening_2mmiso	*Sequence*	diff_dw20_MB
TR	9000 ms	*TR*	5700 ms
TE	66,00 ms	*TE*	79,40 ms
Flip angle	90 deg	*Flip angle*	90 deg
Refocusing flip angle	180 deg	*Refocusing flip angle*	180 deg
FOV	240 x 240 mm	*FOV*	240 x 240 mm
Matrix	128 x 128	*Matrix*	160 x 160
Slice thickness	2 mm isotropic, 70 slices	*Slice thickness*	1,5 mm isotropic, 94 slices
Multiband accel. factor	1	*Multiband accel. factor*	2
Echo spacing	0,54 ms	*Echo spacing*	0,65 ms
BW	2192 Hz/Px	*BW*	1838 Hz/Px
Phase partial Fourier	6/8	*Phase partial Fourier*	6/8
b-values	0, 1500 s/mm^2	*b-values*	0, 1500 s/mm^2

Table 4: Acquisition parameters for screening

2.3 T1

Two types of T1 images were acquired:

- High-resolution (0.75mm) anatomical images.

Parameter	Value
Sequence	Anatomy_T1_0.75mm
TR	2300 ms
TE	3.16 ms
Flip angle	9 deg
FOV	240 x 240 mm
Matrix	128x128
Slice thickness	0.75 mm, 224 slices, 0.75 mm isotropic
Multiband accel. factor	1
Echo spacing	7.8 ms
BW	240 Hz/Px
Phase partial Fourier	7/8
b-values	0 s/mm^2

Table 5: Acquisition parameters for high-resolution T1 images

- MPRAGE T1 image in sagittal view.

Parameter	Value
Sequence	mprage_sag_T1_160sl
TR	2300 ms
TE	2.98 ms
Flip angle	9 deg
FOV	256 x 256 mm
Matrix	256 x 256
Slice thickness	1 mm, 160 slices, 1 mm isotropic
Multiband accel. factor	1
Echo spacing	7.1 ms
BW	240 Hz/Px
Phase partial Fourier	7/8
b-values	0 s/mm^2

Table 6: Acquisition parameters for MPRAGE sagittal T1

2.3.1 T1 relaxometry

Three different runs were performed:

- A B1 map for T1 mapping.

Parameter	Value
Sequence	B1Map_for_T1_map
TR	20000 ms
TE	2.59 ms
Flip angle	8 deg
FOV	256 x 256 mm
Matrix	128 x 128
Slice thickness	2 mm, 44 slices, 2 mm isotropic
Multiband accel. factor	1
Echo spacing	4.5 ms
BW	800 Hz/Px
Phase partial Fourier	None
b-values	0 s/mm^2

Table 7: Acquisition parameters for B1 maps

- T1 maps with FA from 3 to 19 in steps of two.

Parameter	Value
Sequence	T1Map_1mm
TR	10 ms
TE	3 ms
Flip angle	3 deg
FOV	256 x 256 mm
Matrix	128 x 128
Slice thickness	1 mm, 176 slices, 1 mm isotropic
Multiband accel. factor	1
BW	240 Hz/Px
Phase partial Fourier	7/8
b-values	0 s/mm^2

Table 8: Acquisition parameters for T1 maps

2.4 T2

Several types of images were acquired under this category:

- T2 SPC sagittal images.

Parameter	Value
Sequence	T2_SPC_SAG_FOV230
TR	3200 ms
TE	419 ms
Flip angle mode	T2 var
FOV	230 x 230 mm
Matrix	256 x 256
Slice thickness	0.90 mm, 176 slices, 0.90 mm isotropic
Multiband accel. factor	1
Echo spacing	3.52 ms
BW	698 Hz/Px
Phase partial Fourier	None
b-values	0 s/mm^2

Table 9: Acquisition parameters for T2 sagittal images

- T2 FLAIR sagittal.

Parameter	Value
Sequence	T2_FLAIR_SAG_FOV230
TR	5000 ms
TE	396 ms
Flip angle mode	T2 var
FOV	230 x 230 mm
Matrix	256 x 256
Slice thickness	0.81 mm, 192 slices, 0.81 mm isotropic
Multiband accel. factor	1
Echo spacing	3,36 ms
BW	781 Hz/Px
Phase partial Fourier	0
b-values	0 s/mm^2

Table 10: Acquisition parameters for T2 FLAIR sagittal images

- T2 sagittal with fat saturation.

Parameter	Value
Sequence	T2_SPC_SAG_fatsat
TR	3200 ms
TE	420 ms
Flip angle mode	T2 var
FOV	270 x 270 mm
Matrix	384 x 384
Slice thickness	0.70 mm, 240 slices, 0.70 mm isotropic
Echo spacing	3.68 ms
BW	723 Hz/Px
Phase partial Fourier	None
b-values	0 s/mm^2

Table 11: Acquisition parameters for T2 images with Fat-Sat

- T2 sagittal (0.7mm).

Parameter	Value
Sequence	T2_SPC_SAG_0.7mm
TR	3200 ms
TE	420 ms
Flip angle mode	T2 var
FOV	270 x 270 mm
Matrix	384 x 384
Slice thickness	0.70 mm, 240 slices, 0.70 mm isotropic
Multiband accel. factor	1
Echo spacing	3.68 ms
BW	723 Hz/Px
Phase partial Fourier	None
b-values	0 s/mm^2

Table 12: Acquisition parameters for high-resolution sagittal T2 images

2.4.1 T2 relaxometry

Two types of relaxometry images were acquired:

- T2* sagittal (relaxometry).

Parameter	Value
Sequence	relaxometry_T2star_sag
TR	50 ms
TE1	1.77 ms
TE2	5.06 ms
TE3	8.35 ms
TE4	11.64 ms
TE5	14.93 ms
TE6	18.22 ms
TE7	21.51 ms
TE8	24.80 ms
TE9	28.09 ms
TE10	32.50 ms
TE11	38.90 ms
TE12	47.00 ms
Flip angle	20 deg
FOV	288 x 288 mm
Matrix	196 x 196
Slice thickness	1.50 mm, 120 slices, 1.50 mm isotropic
Multiband accel. factor	1
BW	420 Hz/Px
Phase partial Fourier	7/8
b-values	0 s/mm^2

Table 13: Acquisition parameters for T2 relaxometry images

- T2 relaxometry with 12 contrasts.

Parameter	Value
Sequence	relaxometry_T2_tra_12contrastes
TR	7600 ms
TE1	14 ms
Flip angle	180 deg
FOV	256 x 256 mm
Matrix	256 x 256
Slice thickness	1,1 mm, 128 slices, 1,1 mm isotropic
GRAPPA accel. factor	3
Echo spacing	14 ms
BW	215 Hz/Px
Phase partial Fourier	None
b-values	0 s/mm^2

Table 14: Acquisition parameters for 12-contrast T2 images

2.5 BOLD-contrast EPI sequences

The same acquisition parameters were used in all runs pertaining the 12 tasks –four ARCHI tasks, seven HCP tasks and the RSVP Language task– that are part of the first IBC-dataset release, except the number of repetitions (TRs) as each run had a different duration. Table 15 contains the acquisition parameters for the ARCHI Standard task. Table 16 contains the number of TRs for every task:

Parameter	Value
Sequence	Archi_STD
TR	2000 ms
TE	27 ms
Flip angle mode	74 def
FOV	192 x 192 mm
Matrix	128 x 128
Slice thickness	1.50 mm, 93 slices, 1.50 mm isotropic
Multiband accel. factor	3
Echo spacing	0,65 ms
BW	1776 Hz/Px
Phase partial Fourier	None
B-values	0 s/mm^2

Table 15: Acquisition parameters for BOLD-contrast images

Task	Repetitions (TR)	Duration: ss (mm:ss)
ARCHI Standard	156	312 (5:12)
ARCHI Spatial	252	504 (8:24)
ARCHI Social	262	524 (8:44)
ARCHI Emotional	220	442 (7:22)
HCP Language	229	458 (7:38)
HCP Emotion	139	278 (4:38)
HCP Gambling	188	376 (6:16)
HCP Motor	185	370 (6:10)
HCP Social	196	392 (6:32)
HCP Relational	311	622 (10:22)
HCP WM	303	606 (10:06)
RVSP Language	310	620 (10:20)

Table 16: Number of repetitions (TR) for each task

3 Tasks

One of the main goals of the IBC project is to provide an open-source and extensive functional atlas of the human brain. Such endeavour calls for probing many different cognitive tasks.

To avoid sources of inter-subject plus inter-site variability across tasks, the IBC project aims at providing an heterogeneous task-fMRI dataset acquired in a fixed environment.

The data are obtained from a permanent cohort of 12 participants during the performance of a dozen of tasks. Given its task-wise organization, the IBC dataset encompasses a wide range of psychological domains that will yield a refined characterization of the neurocognitive mechanisms underlying human behavior.

3.1 ARCHI tasks

The ARCHI tasks are a battery of localizers comprising a wide range of psychological domains. A description about each task and its corresponding conditions is presented over the next sections.

3.1.1 ARCHI Standard

Paradigm: fast event-related design
Duration: 307s

This task, described in [Pinel et al., 2007] probes basic functions, such as button presses with the left or right hand, viewing horizontal and vertical checkerboards, reading and listening to short sentences, and mental computations (subtractions). Visual stimuli were displayed in four 250-ms epochs, separated by 100ms intervals (i.e., 1.3s in total). Auditory stimuli were generated from a recorded male voice (i.e., a total of 1.6s for motor instructions, 1.2-1.7s for sentences, and 1.2-1.3s for subtraction). The auditory or visual stimuli were shown to the participants for passive viewing or button response in event-related paradigms. Informal inquiries undertaken after the MRI session confirmed that the experimental tasks were understood and followed correctly. This task comprises 10 conditions described in Table 17 and represented in Figure 3.

Condition	Description
• *audio left-button press*	left-hand three-times button press, indicated by auditory instruction
• *audio right-button press*	right-hand three-times button press, indicated by auditory instruction
• *video left-button press*	left-hand three-times button press, indicated by visual instruction
• *video right-button press*	right-hand three-times button press, indicated by visual instruction
• *horizontal checkerboard*	visualization of flashing horizontal checkerboards
• *vertical checkerboard*	visualization of flashing vertical checkerboards
• *sentence listening*	listen to narrative sentences
• *sentence reading*	read narrative sentences
• *audio subtraction*	mental subtraction, indicated by auditory instruction
• *video subtraction*	mental subtraction, indicated by visual instruction

Table 17: Conditions for the ARCHI Standard task

The contrasts derived from the conditions of this task are described in Table 18.

Contrast id	Specification
• *left – right button press*	left hand vs. right hand
• *reading – listening*	sentence reading vs. sentence listening
• *motor – cognitive*	button presses vs. narrative/computation
• *reading – checkerboard*	sentence reading vs. checkerboard
• *computation – sentences*	mental subtraction vs. sentence reading
• *horizontal – vertical*	horizontal checkerboard vs. vertical checkerboard

Table 18: Contrasts for the ARCHI Standard task

3.1.2 ARCHI Spatial

Paradigm: block-design (block duration: 5 to 7s)
Duration: 489s

This task includes the performance of *(1)* ocular saccade, *(2)* grasping and *(3)* orientation judgments on objects (the two different tasks were actually made on the same visual stimuli in order to characterize grasping-specific activity), *(4)* judging whether a hand photograph was the left or right hand or *(5)* was displaying the front or back. The same input stimuli were presented twice in order to characterize specific reponse to hand side judgment. This task comprises 5 conditions described in Table 19 and represented in Figure 4.

Condition	Description
• *saccades*	ocular movements were performed according to the displacement of a fixation cross from the center towards peripheral points in the image displayed
• *left or right hand*	mental judgment on whether the hand displayed on the image is a left or a right hand
• *hand palm or back*	mental judgment on the palmar-dorsal direction of a hand displayed as visual stimulus
• *object grasping*	mimicry of object grasping with right hand, in which the corresponding object was displayed on the screen
• *orientation judgment*	mimic orientation of rhombus, displayed as image background on the screen[1], using right hand along with fingers

Table 19: Conditions for the ARCHI Spatial task

The contrasts derived from the conditions of this task are described in Table 20.

Contrast id	Specification
• *saccades*	saccades vs. fixation
• *hand – side*	left or right hand vs. hand palm or back
• *grasp – orientation*	object grasping vs. orientation judgment
• *rotation side*	rotation side vs. fixation
• *object orientation*	object orientation vs. fixation

Table 20: Contrasts for the ARCHI Spatial task

3.1.3 ARCHI Social

Paradigm: block-design (block duration: 5 to 7s)
Duration: 516s

This task relies on *(1)* the interpretation of short stories involving false beliefs or not, *(2)* observation of moving objects with or without a putative intention, and *(3)* listening to speech and non-speech sounds. The conditions are described in Table 21 and represented in Figure 5.

Condition	Description
• *mechanistic tale*	interpret short stories (presented as auditory stimuli) through mental reply (no active response was involved), featuring a cause-consequence plot
• *mechanistic story*	interpret short stories (presented as visual stimuli) through mental reply (no active response was involved), featuring a cause-consequence plot
• *false-belief tale*	interpret short stories (presented as auditory stimuli) through mental reply (no active response was involved), featuring a *false-belief* plot
• *false-belief story*	interpret short stories (presented as visual stimuli) through mental reply (no active response was involved), featuring a *false-belief* plot
• *social-interaction motion*	watch short movies of triangles, which exhibit a putative interaction
• *random motion*	watch short movies of triangles, which exhibit a random movement
• *voice sound*	listen passively to short samples of human voices
• *natural sound*	listen passively to short samples of natural sounds

Table 21: Conditions for the ARCHI Social task

The contrasts derived from the conditions of this task are described in Table 22.

Contrast id	Specification
• *mechanistic audio*	listening to a mechanistic tale
• *mechanistic video*	reading a mechanistic story
• *false belief – mechanistic audio*	false-belief tale vs. mechanistic tale (audio)
• *false belief – mechanistic video*	false-belief story vs. mechanistic story (video)
• *triangle mental – random*	social-interaction motion vs. random motion
• *triangle random*	randomly drifting figures
• *non speech sound*	listen to natural sound
• *speech sound – non speech sound*	listen to voice sound vs. natural sound

Table 22: Contrasts for the ARCHI Social task

3.1.4 ARCHI Emotional

Paradigm: block-design (block duration: 5 to 7s)
Duration: 436s

This task includes *(1)* facial judgments of gender, and *(2)* trustworthiness plus expression based on complete portraits or photos of eyes' expressions. The conditions are described in Table 23 and represented in Figure 6.

Condition	Description
• *gender assessment of face*	gender evaluation of the presented human faces
• *face control*	mental assessment on the slope of a gray-scale grid image (obtained from scrambling a face's image) that may be tilted or not
• *face trusty*	trustworthy evaluation of the presented human faces
• *expression intention*	trustworthy evaluation of the presented human eye images
• *gender assessment of eye's expression*	gender evaluation of the presented human eye images
• *expression control*	mental assessment on the slope of a gray- scale grid image (obtained from scrambling an eyes' image)that may be tilted or not

Table 23: Conditions for the ARCHI Emotional task

The contrasts derived from the conditions of this task are described in Table 24.

Contrast id	Specification
• *expression gender – control*	gender assessment from eyes' expression
• *expression intention – gender*	expression intention vs. gender assessment
• *face gender – control*	gender assessment from face image
• *face trusty – gender*	face trusty vs. gender assessment

Table 24: Contrasts for the ARCHI Emotional task

3.2 HCP tasks

The HCP tasks used herein were reproductions made in a subset of task-fMRI paradigms originally developed for the Human Connectome Project (HCP, [Barch et al., 2013]), but with minor changes. The adjustments mainly concerned to the translation of all stimuli plus instructions into french, the increment of the number of blocks[2] or the amount of events within blocks[3], among other technical adaptations with respect to the environment in the scanner. No conceptual modifications on the tasks and alterations in the temporal sequence of the conditions were undertaken.

Data from each task were acquired in two runs, within the same session and using different phase-encoding directions.

For sake of clarity, a short description about conditions in the applied tasks is briefly presented over the next sections. Table 25 summarizes all conditions for this set of tasks.

3.2.1 HCP Emotion

The main purpose of *HCP Emotion* task was to capture neural activity arising from fear- or angry-response processes. To elicit stronger effects, affective facial expressions were used as visual stimuli due to their importance in adaptive social behavior [Hariri et al., 2002].

The paradigm was thus composed by two categories of blocks: *(1)* the face block, and *(2)* the shape block. All blocks consisted of a series of events, in which images with faces or shapes were displayed, respectively. There were always three faces/shapes per image; one face/shape was shown at the top and two faces/shapes were shown at the bottom. The participants were then asked to decide which face/shape at the bottom, i.e. left or right face/shape, matched the one displayed at the top, by pressing the corresponding button of the response box.

The task was formed by twelve blocks per run, i.e. six face blocks and six shape blocks. The two block categories were alternately presented for each run. All blocks

[2]The number of blocks was doubled in the majority of the tasks.
[3]This adjustement was only performed for the HCP Relational task.

contained six trials and they were always initiated by a cue of three seconds. In turn, the trials included a visual-stimulus period of two seconds and a fixation-cross period of one second; the total duration of the trial was thus three seconds. The experimental design of this task is represented in Figure 7.

3.2.2 HCP Gambling

This task was adapted from the *Incentive processing* task-fMRI paradigm of the HCP and its aim was to localize brain structures that take part to the reward system, namely the basal ganglia complex.

The paradigm included eight blocks and each block was composed by eight events. For every event, the participants were asked to play a game. The goal was to guess whether the next number to be displayed, which ranged from one to nine, would be more or less than five while a question mark was shown on the screen. The answer was given by pressing the respective button of the response box. Feedback on the correct number was provided afterwards. There was an equal amount of blocks in which the participants experienced either *reward* or *loss*, for most of the events. Concretely, six out of the eight events within a block pertained to one of these two outcomes; the remaining events corresponded to the antagonist or a neutral outcome, i.e. when the correct number was five.

The task was constituted by eight blocks per run, in which each half related to reward and loss experience, respectively. The order of the two block categories were pseudo-randomized during a single run, but fixed for all participants. A fixation-cross period of fifteen seconds was displayed between blocks. All blocks contained eight trials. The trials included a question-mark visual stimulus lasting up to 1.5 seconds, a feedback period of one second and a fixation-cross period of one second, as well; the total duration of the trial was then 3.5 seconds, approximately. The experimental design of this task is represented in Figure 8.

3.2.3 HCP Motor

HCP Motor task was designed with the intent of extracting maps on gross motor topography, in particular motor skills associated with movements of the foot, hand and tongue.

There were thus five categories of blocks with respect to motor tasks involving *(1)* the left foot, *(2)* the right foot, *(3)* the left hand, *(4)* the right hand, and *(5)* the tongue, respectively. The blocks always started with visual cues referring to which part of the body should be moved. The cues were then followed by a set of events, which were in turn indicated by flashing arrows on the screen. The events pertained to the corresponding movements performed by the participants.

The task was formed by five blocks per category, with a total of twenty blocks per run. The order of the block categories were pseudo-randomized during each run, but

fixed for all participants. A fixation-dot period of fifteen seconds was inserted between some blocks. All blocks contained ten trials. Every trial included a cue of one second and a period of performance of twelve seconds[4]; the total duration of the trial was then thirteen seconds. The experimental design of this task is represented in Figure 9.

3.2.4 HCP Language

HCP Language task was used as a localizer of brain regions involved in semantic processing, with special focus on the anterior temporal lobe (ATL) [Binder et al., 2011].

The paradigm comprised two categories of blocks: *(1)* story blocks, and *(2)* math blocks. The math block served as a control task in this context, since it was likely to adress other brain regions during the attentional demands. Both type of blocks exhibited auditory stimuli in short epochs, which in turn finished with a final question followed by two possible answers. During story blocks, participants were presented with stories, whose question targeted their respective topics. Conversely, math blocks showed arithmetic problems for which the correct solution must be selected. The answer was provided after the two possible options were displayed, through pressing the corresponding button of the response box. The difficulty levels of the problems, presented for both categories, were adjusted throughout the experiment, in order to keep the participants engaged in the task and, thus, assure accurate performances [Binder et al., 2011].

The task was composed by eleven blocks per run. For the first run, six story blocks and five math blocks were interleaved, respectively. The reverse amount and order of blocks were used during the second run. The number of trials per block varied between one and four. Nevertheless, it was assured that both block categories matched their length of presentation at every run. There was a cue of two seconds in the beginning of each block, indicating its category. The duration of the trials within a block varied between ten and thirty seconds. Finally, the presentation of the auditory stimuli was always accompanied by the display of a fixation cross on the screen throughout the entire run. The experimental design of this task is represented in Figure 10.

3.2.5 HCP Relational

HCP Relational task employed a relational matching-to-sample paradigm, featuring a second-order comparison of relations between two pairs of objects. It served primarily as a localizer of the rostrolateral prefrontal cortex, since *relational matching* mechanisms were shown to elicit activation on this region [Smith et al., 2007].

Similarly to some previous tasks, two categories of blocks described the paradigm: *(1)* the *relational-processing* block, and *(2)* the *control-matching* block. All blocks were constituted by a set of events. In the relational-processing block, visual stimuli consisted of images representing two pairs of objects, in which one pair was placed at the top and

[4] During the period of performance, arrows flashed ten times on the screen, as an indication of the number of movements that should be performed.

the other one at the bottom of the image, respectively. Objects within a pair may differ in two dimensions: shape and texture. The participants had to identify whether the pair of objects from the top differed in a specific dimension and, subsequently, they were asked to determine whether the pair from the bottom changed along the same dimension. For the control block, one pair of objects was displayed at the top of the image and a single object at the bottom of the same image. In addition, a cue was shown in the middle of that image referring to one of the two possible dimensions. The participants had thus to indicate whether the object from the bottom was matching either of the two objects from the top, according to the dimension specified as a cue.

This task was formed by twelve blocks per run. Two groups of six blocks referred to the two block categories, respectively. Block categories were, in turn, interleaved for display within a run. A fixation-cross period of sixteen seconds was inserted between some blocks. All blocks contained six trials and they were always initiated by a cue of two seconds. The trials were described by a visual-stimulus plus response period followed by a fixation-cross period, lasting up to ten seconds. The duration of the former differed in agreement with the type of block, i.e. it lasted nine seconds and 7.6 seconds during the relational-processing block and control-matching block, respectively. The experimental design of this task is represented in Figure 11.

3.2.6 HCP Social

HCP Social task intended to provide evidence for task-specific activation in brain structures presumably implicated in social cognition.

The paradigm included two categories of blocks, in which movies were presented during short epochs. The movies consisted in triangle-shape clip art, moving in a predetermined fashion. Putative social interactions could be drawn from movements referring to the block category on the effect-of-interest. In contrast, objects appeared to be randomly moving the other category, i.e. the control-effect block.

The task was constituted by ten blocks per run. Each half of the blocks corresponded to one of the aforementioned block categories, whose order was pseudo-randomized for every run, but fixed for all participants. There was only one trial present per block. It consisted of a twenty-second period of video-clip presentation plus three seconds maximum of a response period, indicated by a momentary instruction on the screen. Thus, the total duration of a block was approximately twenty three seconds. A fixation-cross period of fifteen seconds was always displayed between blocks. The experimental design of this task is represented in Figure 12.

3.2.7 HCP Working Memory

HCP Working Memory task was adapted from the classical *n-back* task to serve as functional localizer for evaluation of working-memory (WM) capacity and related processes.

The paradigm integrated two categories of blocks: *(1)* the "0-back" WM-task block, and *(2)* the "2-back" WM-task block. They were both equally presented within a run. A cue was always displayed at the beginning of each block, indicating its task-related type.

Blocks were formed by set of events, during which pictures of faces, places, tools or body parts were shown on the screen. One block was always dedicated to one specific category of pictures and the four categories were always presented at every run.

The task was constituted by sixteen blocks per run, splitted into two block categories. Besides, there were four pairs of blocks per category, referring respectively to the four classes of pictures mentioned above. The order of the blocks, regardless their category and corresponding class of pictures, was pseudo-randomized for every run, but fixed for all participants. A fixation-cross period of fifteen seconds was introduced between some blocks. All blocks contained ten trials and they were always initiated by a cue of 2.5 seconds. Trials included in turn the presentation of a picture for two seconds and a very short fixation-cross period for half of a second; the total duration of one trial was thus 2.5 seconds. The experimental design of this task is represented in Figure 13.

The conditions of the HCP battery of tasks are described in Table 25.

Task	Conditions	Task	Conditions
HCP Language	story	*HCP Social*	social-interaction motion
	math		random motion
HCP Emotion	shape outline	*HCP Relational*	relational processing
	face image		visual matching
HCP Gambling	punishment	*HCP WM*	body image 0-back
	reward		body image 2-back
HCP Motor	left hand		face image 0-back
	right hand		face image 2-back
	left foot		tool image 0-back
	right foot		tool image 2-back
	tongue		place image 0-back
	cue		place image 2-back

Table 25: Conditions for the HCP tasks

The contrasts derived from the conditions of this task are described in Table 26.

Task	Contrast id	Specification
HCP Language	story – math	story vs. math
	math	mental additions
HCP Social	mental – random	social-interaction motion vs. random motion
	random	random motion vs. fixation
HCP Emotion	face – shape	face image vs. shape outline
	shape	shape comparison
HCP Relational	relational – match	relational comparison vs. matching
	match	visual feature matching
HCP Gambling	punishment – reward	positive vs. negative gambling outcome
	reward	gambling with positive outcome
HCP WM	2back – 0back	2-back vs. 0-back
	body – avg	body image vs. any motion
	face – avg	face image vs. any motion
	place – avg	place image vs. any motion
	tools – avg	tool image vs. any motion
HCP Motor	left hand – avg	left hand vs. any motion
	right hand – avg	right hand vs. any motion
	left foot – avg	left foot vs. any motion
	right foot – avg	right foot vs. any motion
	tongue – avg	tongue vs. any motion

Table 26: Contrasts for the HCP tasks

3.3 RSVP Language task

The *Rapid-Serial-Visual-Presentation (RSVP) Language* task was adapted from the study undertaken by [Humphries et al., 2006] on syntactic and semantic processing during auditory sentence comprehension. Specifically, the task herein described targeted the same syntactic and semantic modules, but in the context of reading. It thus allowed for capturing further associations with regard to e.g. visual (pseudo) word recognition and sublexical route, among other aspects related to active reading.

The paradigm consisted in a block-design presentation strategy of the stimuli. One block was defined as an epoch within a trial and epochs corresponded in turn to experimental conditions. Such conditions stood for the consecutive visual presentation of ten constituents composed by letters. There were six different conditions; they are described in Table 27.

All linguistic content elicited from the conditions except "consonant strings", such as grammar rules, lexicon and phonemes, were part of the french language. In order to ensure continuous engagement during task performance, participants were asked, straight

Condition	Description
• *simple sentence*	constituents, i.e. words, formed syntactically and semantically congruent sentences of one single clause (low_sentence-structure_complexity)
• *complex sentence*	constituents, i.e words, formed syntactically and semantically congruent sentences with more than one clause grid image that may be tilted or not (high sentence-structure complexity)
• *read jabberwocky*	syntactically congruent sentences composed by non-lexical vocable constituents
• *read words*	syntactically non-congruent sentences but with semantic content
• *read pseudowords*	syntactically and semantically non-congruent sentences composed by non-lexical vocable constituents
• *consonant strings*	syntactically and semantically non-congruent sentences composed by non-vocable constituents

Table 27: Conditions for the RSVP Language task

afterwards the visualization of every sentence, to ascertain whether the current constituent displayed on the screen, *aka* "the probe", was part of the previous sentence or not. The corresponding answer was provided immediately after the probe, by pressing the respective button of the response box.

Data were collected in six runs during one single session. Every run was composed by sixty trials, in which subsets of ten trials were dedicated to each condition, respectively. The order of the trials was pseudo-randomized within and between runs, such that there were no repeated trials during a full session. Moreover, a different pseudo-randomized order for the presentation of the trials was always employed across participants. One trial comprised several experimental manipulations, other than a block integrating one specific condition. It was sequentially formed by a period of fixation-cross display (two seconds), another short period of a blank screen (0.5 seconds), a block containing the linguistic stimuli (0.4 seconds $\times$ 10 = 4 seconds), a jittered blank screen (varying from one to 1.5 seconds), a period of a second fixation-cross display (0.5 seconds), a period for the probe display (0.5 seconds), and, finally, a response period (varying up to two seconds). The total duration of one single trial was thus ten seconds. Three extra seconds of blank screen were added at the beginning of every run, i.e. before the presentation of the first trial. The experimental design of this task is represented in Figure 14.

Two opposite phase-encoding directions were respectively applied during acquisition of each half of the total amount of runs.

The contrasts derived from the conditions of this task are described in Table 28.

Contrast id	Specification
• *complex – simple*	read sentence with complex vs. simple syntax
• *sentence – jabberwocky*	read sentence vs. read *jabberwocky*
• *sentence – word*	read sentence vs. read words
• *word – consonant string*	read words vs. consonant strings
• *jabberwocky – pseudo*	read *jabberwocky* vs. read pseudowords
• *word – pseudo*	read words vs. read pseudowords
• *pseudo – consonant string*	read pseudowords vs. consonant strings
• *consonant string*	read and encode consonant strings

Table 28: Contrasts for the RSVP Language task

4 Processing pipeline

4.1 Preprocessing pipeline

Source data were preprocessed using *PyPreprocess*. This library offers a collection of Python tools to facilitate pipeline runs, reporting and quality check (https://github.com/neurospin/pypreprocess). It is built upon the *Nipype* library [Gorgolewski et al., 2011] v0.12.1, that in turn launched various commands used to process neuroimaging data. These commands were taken from the *SPM12* software package (Wellcome Department of Imaging Neuroscience, London, UK) v6685, and the *FSL* library (Analysis Group, FMRIB, Oxford, UK) v5.0.

All fMRI images, i.e. GE-EPI volumes, were collected twice with reversed phase-encoding directions, resulting in pairs of images with distortions going in opposite directions. Susceptibility-induced off-resonance field was estimated from the two Spin-Echo EPI volumes in reversed phase-encoding directions. The images were corrected based on the estimated deformation model, using the *topup* tool [Andersson et al., 2003] implemented in FSL [Smith et al., 2004].

Further, the GE-EPI volumes were aligned to each other within each participant. A rigid body transformation was employed, in which the average volume of all images was used as reference [Friston et al., 1995]. The mean EPI volume was also co-registered onto the corresponding T1-weighted MPRAGE (anatomical) volume for every participant [Ashburner and Friston, 1997]. The individual anatomical volumes were then segmented into tissue types to finally allow for the normalization of both anatomical and functional data [Ashburner and Friston, 2005]. Concretely, the segmented volumes were used to compute the deformation field for normalization to the standard MNI152 space. The deformation field was then applied to the EPI data. In the end, all volumes were resampled to their original resolution, i.e. 1 mm isotropic for the T1-weighted MPRAGE images and 1.5 mm for the EPI images.

4.1.1 FMRI Model Specification

The fMRI data were analyzed using the *General Linear Model* (GLM). Regressors of the model were designed to capture variations in BOLD response strictly following stimulus timing specifications. They were estimated through the convolution of temporal representations referring to the task-conditions with the canonical *Hemodynamic Response Function* (HRF), defined according to [Friston et al., 1998a] and [Friston et al., 1998b].

The temporal profile of the conditions was characterized by boxcar functions. To build such models, paradigm descriptors grouped in triplets (i.e. onset time, duration and trial type according to BIDS Specification) were determined from the log files' registries generated by the stimulus-delivery software.

To account for small fluctuations in the latency of the HRF peak response, additional regressors were computed based on the convolution of the same task-conditions profile with the time derivative of the HRF.

Nuisance regressors were also added to the design matrix in order to minimize the final residual error. To remove signal variance associated with spurious effects arising from movements, six temporal regressors were defined for the motion parameters. Further, the first five principal components of the signal, extracted from voxels showing the 5% highest variance, were also regressed to capture physiological noise [Behzadi et al., 2007].

In addition, a discrete-cosine transform set was applied for high-pass filtering (cutoff=128 seconds). Model specification was implemented using *Nistats* library v0.0.1b, a Python module devoted to statistical analysis of fMRI data (`https://nistats.github.io`), which leverages *Nilearn* [Abraham et al., 2014], a Python library for statistical learning on neuroimaging data (`https://nilearn.github.io/`).

4.1.2 Model Estimation

In order to restrict GLM parameters estimation to voxels inside functional brain regions, a brain mask was extracted from the mean EPI volume. The procedure implemented in the Nilearn software simply thresholds the mean fMRI image of each subject in order to separate brain tissue from background, and performs then a morphological opening of the resulting image to remove spurious voxels.

Regarding noise modeling, a first-order autoregressive model was used in the maximum likelihood estimation procedure.

A mass-univariate GLM fit was applied separately to the preprocessed GE-EPI data of each run with respect to a specific task. Parameter estimates pertaining to the experimental conditions were thus computed, along with the respective covariance at every voxel. Various contrasts (linear combinations of the effects), were then defined, referring only to differences in evoked responses between either *(i)* two conditions-of-interest or *(ii)* one condition-of-interest and baseline. GLM estimation and subsequent statistical analyses were also implemented using Nistats v0.1. fMRI data analysis was first run on unsmoothed data and, afterwards, on data smoothed with a 5mm full-width-at-half-maximum kernel. Such procedure allows for increased *Signal-to-Noise Ratio* (SNR) and it facilitates between-image comparison.

References

[Abraham et al., 2014] Abraham, A., Pedregosa, F., Eickenberg, M., Gervais, P., Mueller, A., Kossaifi, J., Gramfort, A., Thirion, B., and Varoquaux, G. (2014). Machine learning for neuroimaging with scikit-learn. *Frontiers in Neuroinformatics*, 8:14.

[Andersson et al., 2003] Andersson, J. L., Skare, S., and Ashburner, J. (2003). How to correct susceptibility distortions in spin-echo echo-planar images: application to diffusion tensor imaging. *NeuroImage*, 20(2):870 – 888.

[Ashburner and Friston, 1997] Ashburner, J. and Friston, K. (1997). Multimodal Image Coregistration and Partitioning - A Unified Framework. *NeuroImage*, 6(3):209–217.

[Ashburner and Friston, 2005] Ashburner, J. and Friston, K. J. (2005). Unified segmentation. *NeuroImage*, 26(3):839–851.

[Barch et al., 2013] Barch, D. M., Burgess, G. C., Harms, M. P., Petersen, S. E., Schlaggar, B. L., Corbetta, M., Glasser, M. F., Curtiss, S., Dixit, S., Feldt, C., Nolan, D., Bryant, E., Hartley, T., Footer, O., Bjork, J. M., Poldrack, R., Smith, S., Johansen-Berg, H., Snyder, A. Z., and Van Essen, D. C. (2013). Function in the human connectome: Task-fMRI and individual differences in behavior. *NeuroImage*, 80:169–89.

[Behzadi et al., 2007] Behzadi, Y., Restom, K., Liau, J., and Liu, T. T. (2007). A component based noise correction method (compcor) for {BOLD} and perfusion based fMRI. *NeuroImage*, 37(1):90 – 101.

[Binder et al., 2011] Binder, J. R., Gross, W. L., Allendorfer, J. B., Bonilha, L., Chapin, J., Edwards, J. C., Grabowski, T. J., Langfitt, J. T., Loring, D. W., Lowe, M. J., Koenig, K., Morgan, P. S., Ojemann, J. G., Rorden, C., Szaflarski, J. P., Tivarus, M. E., and Weaver, K. E. (2011). Mapping anterior temporal lobe language areas with fMRI: A multicenter normative study. *NeuroImage*, 54(2):1465 – 1475.

[Friston et al., 1998a] Friston, K., Fletcher, P., Josephs, O., Holmes, A., Rugg, M., and Turner, R. (1998a). Event-related fMRI: Characterizing differential responses. *NeuroImage*, 7(1):30 – 40.

[Friston et al., 1995] Friston, K. J., Frith, C. D., Frackowiak, R. S. J., and Turner, R. (1995). Characterizing Dynamic Brain Responses with fMRI: a Multivariate Approach. *NeuroImage*, 2(2):166–172.

[Friston et al., 1998b] Friston, K. J., Josephs, O., Rees, G., and Turner, R. (1998b). Nonlinear event-related responses in fMRI. *Magnetic Resonance in Medicine*, 39(1):41–52.

[Gorgolewski et al., 2011] Gorgolewski, K., Burns, C. D., Madison, C., Clark, D., Halchenko, Y. O., Waskom, M. L., and Ghosh, S. S. (2011). Nipype: A flexible, lightweight and extensible neuroimaging data processing framework in python. *Frontiers in Neuroinformatics*, 5.

[Hariri et al., 2002] Hariri, A. R., Tessitore, A., Mattay, V. S., Fera, F., and Weinberger, D. R. (2002). The amygdala response to emotional stimuli: A comparison of faces and scenes. *NeuroImage*, 17(1):317 – 323.

[Humphries et al., 2006] Humphries, C., Binder, J. R., Medler, D. A., and Liebenthal, E. (2006). Syntactic and semantic modulation of neural activity during auditory sentence comprehension. *J. Cognitive Neuroscience*, 18(4):665–679.

[Pinel et al., 2007] Pinel, P., Thirion, B., Meriaux, S., Jobert, A., Serres, J., Bihan, D. L., Poline, J.-B., and Dehaene, S. (2007). Fast reproducible identification and large-scale databasing of individual functional cognitive networks. *BMC Neurosci*, 8:91.

[Smith et al., 2007] Smith, R., Keramatian, K., and Christoff, K. (2007). Localizing the rostrolateral prefrontal cortex at the individual level. *NeuroImage*, 36(4):1387 – 1396.

[Smith et al., 2004] Smith, S. M., Jenkinson, M., Woolrich, M. W., Beckmann, C. F., Behrens, T. E., Johansen-Berg, H., Bannister, P. R., Luca, M. D., Drobnjak, I., Flitney, D. E., Niazy, R. K., Saunders, J., Vickers, J., Zhang, Y., Stefano, N. D., Brady, J. M., and Matthews, P. M. (2004). Advances in functional and structural {MR} image analysis and implementation as {FSL}. *NeuroImage*, 23, Supplement 1:S208 – S219. Mathematics in Brain Imaging.

Appendix A Experimental-design diagrams

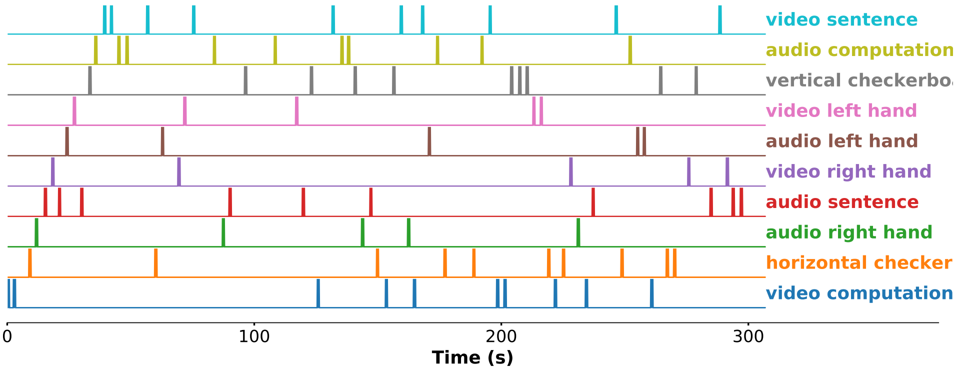

Figure 3: Fast event-related design of the ARCHI Standard task

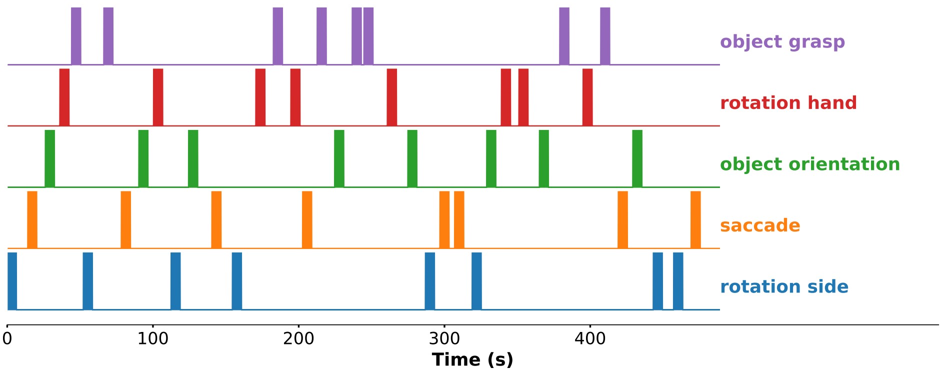

Figure 4: Block-design of the ARCHI Spatial task

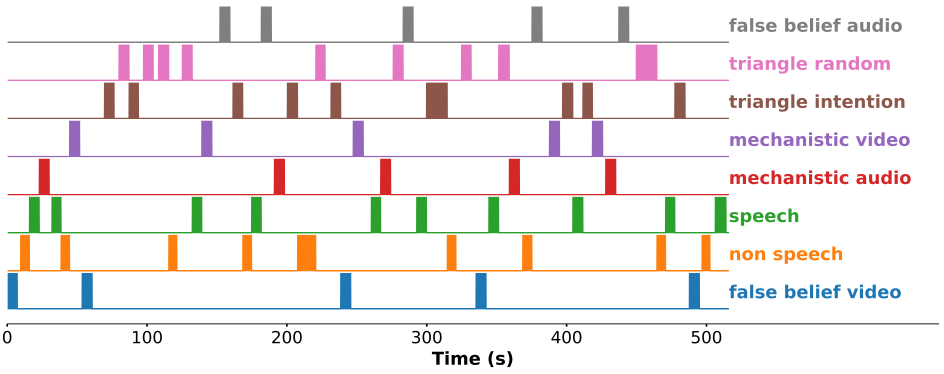

Figure 5: Block-design of the ARCHI Social task

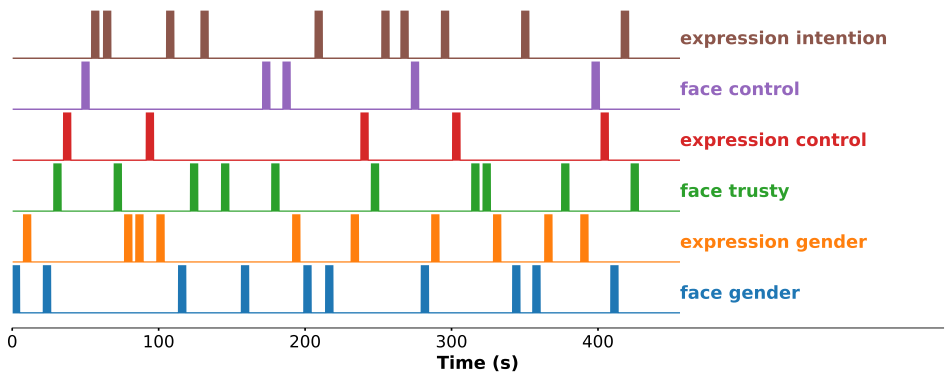

Figure 6: Block-design of the ARCHI Emotional task

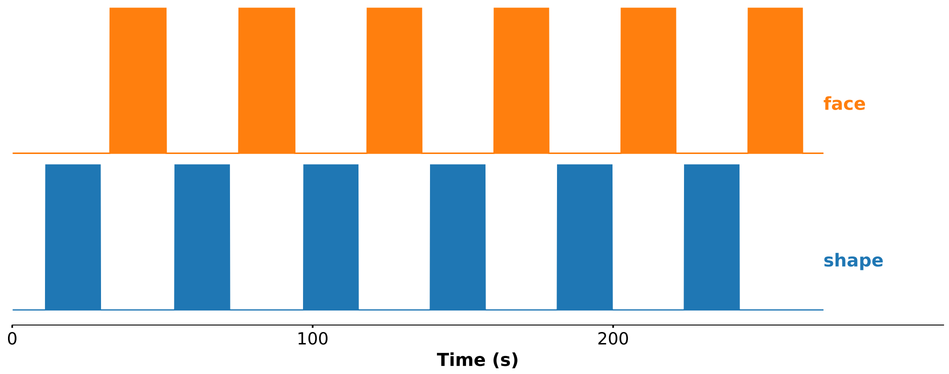

Figure 7: Block-design of the HCP Emotion task

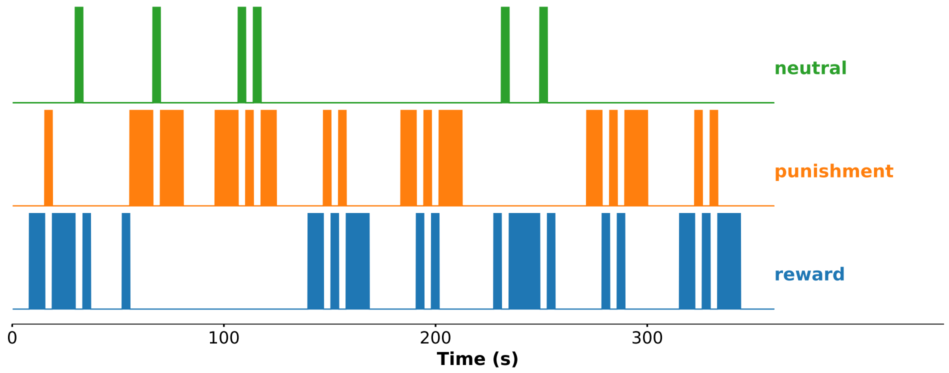

Figure 8: Block-design of the HCP Gambling task

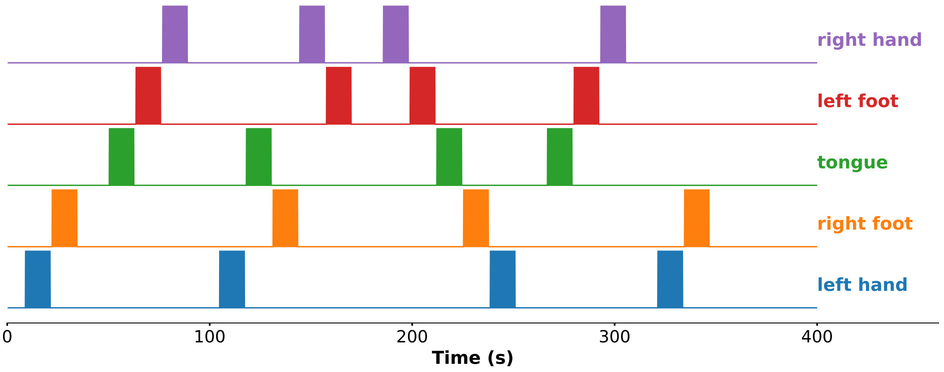

Figure 9: Block-design of the HCP Motor task

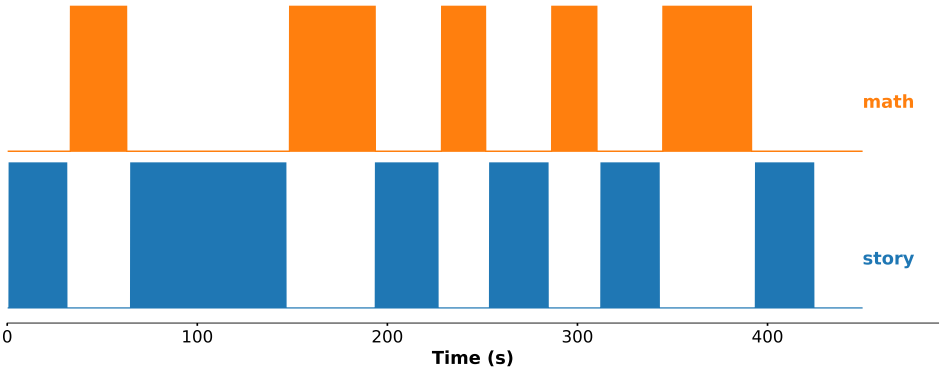

Figure 10: Block-design of the HCP Language task

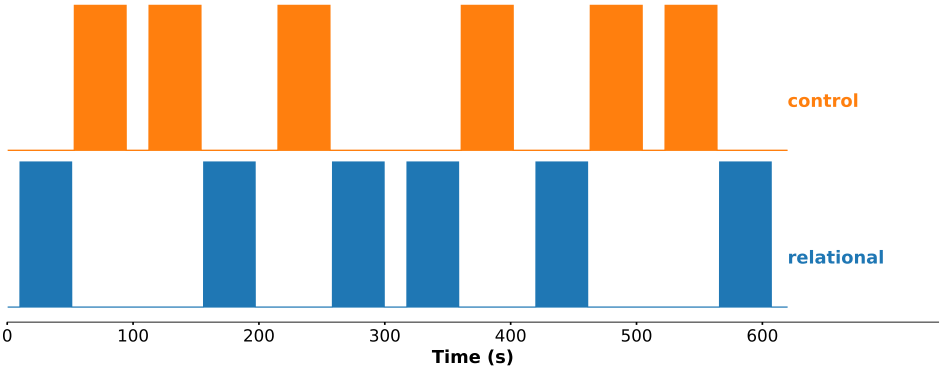

Figure 11: Block-design of the HCP Relational task

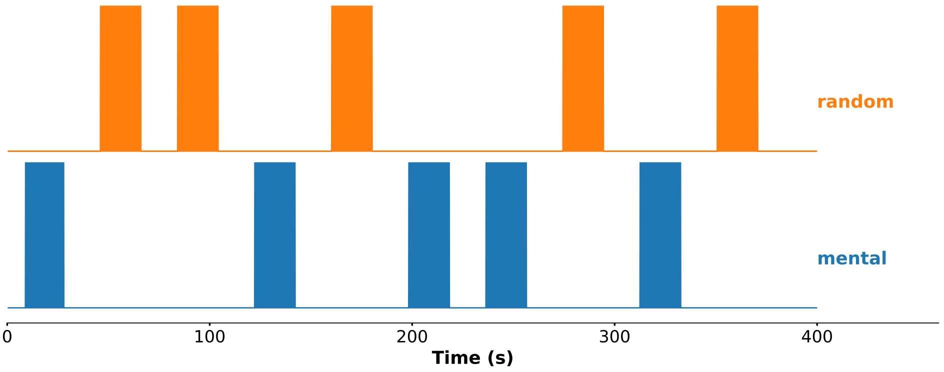

Figure 12: Block-design of the HCP Social task

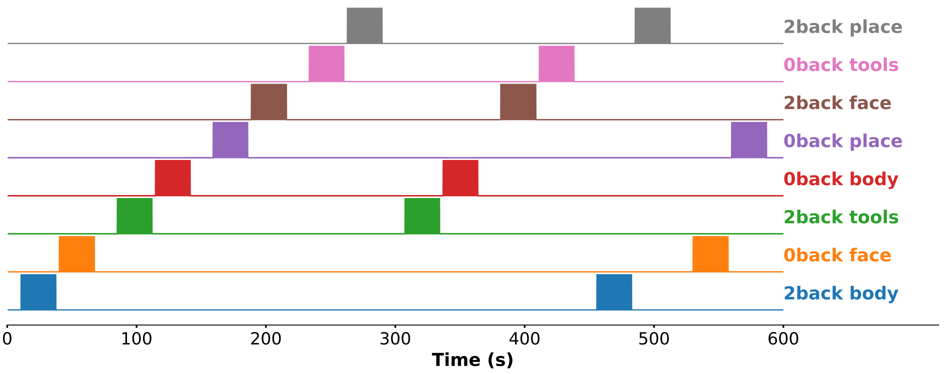

Figure 13: Block-design of the HCP Working-Memory task

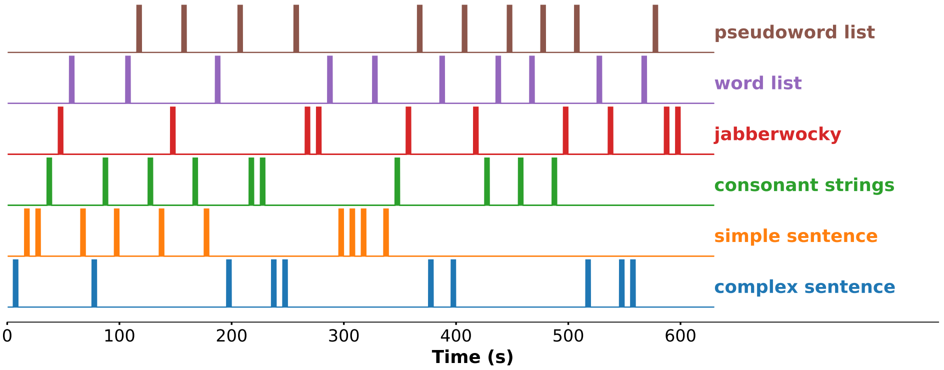

Figure 14: Block-design of the RSVP Language task

Appendix B Acquisition table

	**sub-01**	**sub-02**	**sub-04**	**sub-05**	**sub-06**	**sub-07**	**sub-08**	**sub-09**	**sub-11**	**sub-12**	**sub-13**	**sub-14**	**sub-15**
ses-00	screening	screening	screening	screening	screening	screening	screening	screening	screening	screening	screening	screening	screening
ses-01	old_anat1	archi	hcp1	hcp1	hcp1	hcp1	archi	hcp1	hcp1	hcp1	hcp1	archi	archi
ses-02	old_anat2	old_anat1	hcp2	hcp2	hcp2	hcp2	hcp1	hcp2	hcp2	hcp2	hcp2	hcp1	hcp1
ses-03	hcp1	old_anat2	rsvp-language	rsvp-language	rsvp-language	rsvp-language	hcp2	old_anat1	rsvp-language	archi	rsvp-language	hcp2	hcp2
ses-04	hcp2	hcp1	archi	archi	archi	archi	rsvp-language	rsvp-language	clips1	rsvp-language	archi	rsvp-language	rsvp-language
ses-05	rsvp-language	hcp2	clips1	clips1	clips1	clips1	clips1	archi	archi	clips1	clips1	anat1	tom
ses-06	clips1	rsvp-language	clips2	clips2	clips2	clips2	clips2	clips1	clips2	clips2	clips2	anat2	clips1
ses-07	archi	clips1	clips3	clips3	clips3	clips3	clips3	clips2	clips3	clips3	clips3	clips1	clips2

Table 29: Tasks per participant and session
